# Validation of Candidate Sleep Disorder Risk Genes Using Zebrafish

**DOI:** 10.3389/fnmol.2022.873520

**Published:** 2022-04-07

**Authors:** Steven Tran, David A. Prober

**Affiliations:** Division of Biology and Biological Engineering, Tianqiao and Chrissy Chen Institute for Neuroscience, California Institute of Technology, Pasadena, CA, United States

**Keywords:** zebrafish, sleep, behavior, GWAS, CRISPR/Cas9, genetic screen

## Abstract

Sleep disorders and chronic sleep disturbances are common and are associated with cardio-metabolic diseases and neuropsychiatric disorders. Several genetic pathways and neuronal mechanisms that regulate sleep have been described in animal models, but the genes underlying human sleep variation and sleep disorders are largely unknown. Identifying these genes is essential in order to develop effective therapies for sleep disorders and their associated comorbidities. To address this unmet health problem, genome-wide association studies (GWAS) have identified numerous genetic variants associated with human sleep traits and sleep disorders. However, in most cases, it is unclear which gene is responsible for a sleep phenotype that is associated with a genetic variant. As a result, it is necessary to experimentally validate candidate genes identified by GWAS using an animal model. Rodents are ill-suited for this endeavor due to their poor amenability to high-throughput sleep assays and the high costs associated with generating, maintaining, and testing large numbers of mutant lines. Zebrafish (*Danio rerio*), an alternative vertebrate model for studying sleep, allows for the rapid and cost-effective generation of mutant lines using the CRISPR/Cas9 system. Numerous zebrafish mutant lines can then be tested in parallel using high-throughput behavioral assays to identify genes whose loss affects sleep. This process identifies a gene associated with each GWAS hit that is likely responsible for the human sleep phenotype. This strategy is a powerful complement to GWAS approaches and holds great promise to identify the genetic basis for common human sleep disorders.

## Introduction

Over 10% of Americans suffer from a clinically significant sleep disorder (Ram et al., 2010) and 25%–30% of adults worldwide report chronic sleep disturbances (Stranges et al., 2012), which can be associated with cardio-metabolic and neuropsychiatric disorders (Luyster et al., 2012; Fernandez-Mendoza and Vgontzas, 2013). In addition, up to 90% of individuals with a mental illness report sleep disturbances (Krystal, 2012), which may exacerbate underlying neurological symptoms of anxiety and related disorders (Cox and Olatunji, 2016). Despite the impact of poor sleep quality on human health, current options for treating sleep disorders are limited, and sleep disorders thus represent an unmet public health problem (Colten and Altevogt, 2006). This limitation is partly due to our poor understanding of genetic and neuronal mechanisms that regulate sleep, resulting in few known therapeutic targets. Thus, identifying genes that underlie human sleep disorders is a critical first step in order to develop novel therapies. Genes that underlie narcolepsy (Mahoney et al., 2019), some circadian sleep disorders (Gentry et al., 2021), and a small number of other sleep disorders (Gentry et al., 2021) have been identified based on studies using humans and animal models, but these disorders represent only a small fraction of commonly reported sleep disturbances. Nevertheless, these discoveries have already led to novel therapies. For example, the discoveries that narcolepsy results from loss of signaling by the neuropeptide hypocretin/orexin (Chemelli et al., 1999; Lin et al., 1999), and that hypocretin/orexin signaling is arousing (Adamantidis et al., 2007), led to the development of hypocretin/orexin receptor antagonists as an effective treatment for insomnia (Sun et al., 2021). Conversely, hypocretin/orexin receptor agonists are currently under development as therapies for narcolepsy (Sun et al., 2021). Most experimental sleep studies use model organisms such as rodents, zebrafish, fruit flies, and nematodes, and these studies have identified several genetic pathways that regulate sleep (Oikonomou and Prober, 2017; Saper and Fuller, 2017; Scammell et al., 2017; Liu and Dan, 2019). However, the relevance of these pathways to human sleep disorders is unclear. An alternative approach is to use human genetic studies to identify candidate genes that may underlie human sleep disorders, and then use animal models to validate these candidate genes, determine how they affect sleep, and develop therapeutics.

## Genome-Wide Association Studies (GWAS) Identify Candidate Genes Associated with Human Sleep Variation and Sleep Disorders

**Figure 1:**
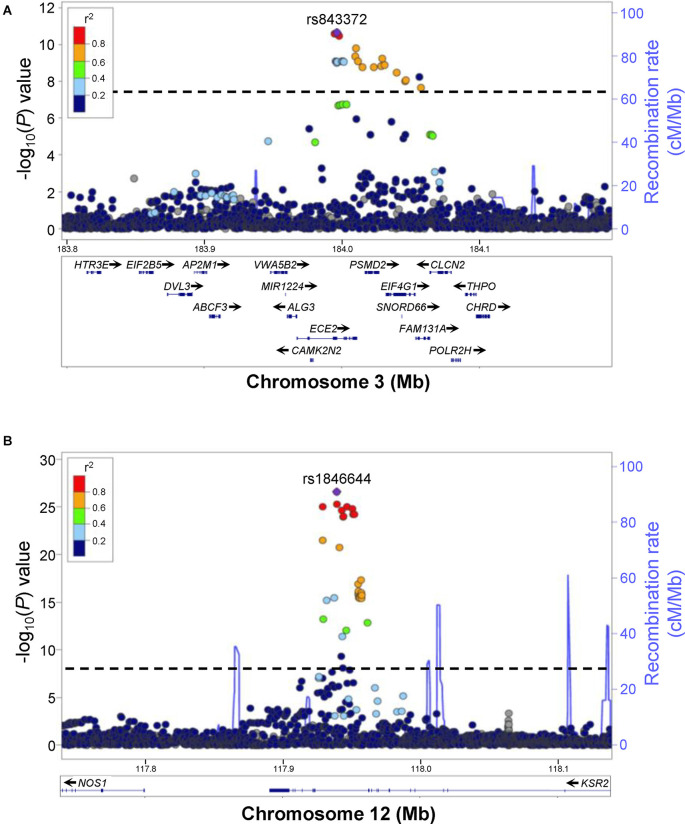
Examples of human GWAS data for excessive daytime sleepiness (EDS). Regional association plots for genome-wide significant associations for rs843372 **(A)** and rs1846644 **(B)** with EDS are shown. Genes within each region are shown in the lower panels. Blue lines indicate recombination rate. Filled circles show the −log_10_ P-value for each SNP, with the named SNP shown in purple. Additional SNPs are colored according to correlation (r^2^) with the lead SNP, estimated by LocusZoom based on the CEU HapMap haplotypes. Dashed black lines indicate the genome-wide significance threshold (*P* < 5 × 10^−8^). **(A)** rs843372 and nearby SNPs that have a genome-wide significant association with EDS are spread out over several different genes, making it difficult to predict which gene is associated with EDS. **(B)** rs1846644 and nearby SNPs that have a genome-wide significant association with EDS are located within introns of the KSR2 gene locus, suggesting that variation in KSR2 might be associated with EDS. Reproduced and modified from Lee et al. (2019).

GWAS aim to identify associations between genetic variants, such as single-nucleotide polymorphisms (SNPs), and human traits or diseases from large population samples (see Uffelmann et al., 2021 for review). By correlating the frequency at which genetic variants are associated with a specific trait, this approach can identify genomic regions associated with that trait, also known as genomic risk loci. Since the introduction of GWAS, numerous genomic risk loci associated with various traits and diseases have been identified, including obesity (Frayling et al., 2007), autoimmune disorders (Siminovitch, 2004), and neuropsychiatric disorders (Schizophrenia Working Group of the Psychiatric Genomics, 2014; Grove et al., 2019; Levey et al., 2021). Genetic insights from some of these studies have led to the development of novel drugs to treat specific diseases (Moschen et al., 2019). Several GWAS that include hundreds of thousands to over a million human subjects have recently been described that analyze both subjective and objective sleep data (Gottlieb et al., 2015; Hu et al., 2016; Jones et al., 2016, 2019; Lane et al., 2016, 2017, 2019; Hammerschlag et al., 2017; Doherty et al., 2018; Dashti et al., 2019, 2021; Jansen et al., 2019; Wang et al., 2019; Garfield, 2021). Most of these studies utilized datasets from large biomedical databases such as the UK Biobank (Bycroft et al., 2018) and 23andMe (Eriksson et al., 2010), which primarily include individuals of European descent. Self-reported sleep traits assayed in these studies include sleep duration, difficulty initiating sleep, difficulty maintaining sleep, and excessive daytime sleepiness. Although there are limitations to self-reported sleep data (Lauderdale et al., 2008; Matthews et al., 2018), the validity of self-reported sleep duration data is supported by studies that use wrist-worn accelerometers (Doherty et al., 2018; Dashti et al., 2019; Jones et al., 2019). These studies have identified a large number of genomic loci associated with human sleep traits and sleep disorders, but in most cases it is unclear which gene within a particular genomic locus is associated with the sleep phenotype. This can be due to the presence of multiple genetic variants that are associated with the same phenotype but overlap with multiple neighboring genes (Figure 1A). In addition, even when only a single gene is located near a genetic variant, the gene responsible for the phenotype can be located in a more distant genomic region (Porcu et al., 2019). For example, SNPs associated with obesity and type-2 diabetes that are located in noncoding regions of the *FTO* gene locus interact with the promoter of the *IRX3* gene at megabase distances, thus affecting the expression of *IRX3*, which regulates body weight in mice (Smemo et al., 2014; Claussnitzer et al., 2015). Thus, while it is often assumed that the gene nearest to a genetic variant is responsible for a phenotype, this is often not the case. Strategies such as heuristic linkage disequilibrium, penalized regression, and Bayesian fine-mapping have been used to first narrow down causal variants and then genes using variant-to-gene mapping (Schaid et al., 2018). More recently, GWAS data and chromatin conformation capture were used for variant-to-gene mapping to identify 88 candidate insomnia risk genes (Palermo et al., 2021). The orthologs of many of these genes were then functionally tested using RNAi knockdown in *Drosophila*, which identified a small number of genes whose loss resulted in a sleep phenotype. The function of one of these genes was tested using CRISPR/Cas9 knockdown in zebrafish, which resulted in a phenotype similar to that observed in *Drosophila*. Thus, while variant-to-gene mapping methods can narrow down a list of candidate genes (Chesi et al., 2019), these candidates must ultimately be validated using an animal model in order to attribute causality to a particular gene. Rodent models have provided many insights into mechanisms that underlie sleep (Saper and Fuller, 2017; Scammell et al., 2017; Liu and Dan, 2019), but are poorly suited for validating large numbers of candidate disease risk genes due to the high cost and labor associated with generating, maintaining, and testing large collections of rodent mutant lines. In contrast, the use of genetics to study sleep was largely pioneered using *Drosophila* (Hendricks et al., 2000; Shaw et al., 2000; Cirelli et al., 2005), which has been used to identify a large number of genes and neuronal circuits that regulate sleep (Allada et al., 2017; Artiushin and Sehgal, 2017). Unlike mice, *Drosophila* are well suited for high-throughput sleep studies that monitor sleep/wake states using beam breaks (Hendricks et al., 2000; Shaw et al., 2000) or, similar to zebrafish, a videotracking system (Guo et al., 2016). RNAi libraries for conditional gene knockdown in specific cell types (Dietzl et al., 2007) are available to quickly test many candidate genes for sleep phenotypes, an approach that has identified genes that affect sleep and the cells in which they function. However, *Drosophila* have several disadvantages as a sleep model. First, there is little anatomical or molecular homology between vertebrate and invertebrate brains (Lichtneckert and Reichert, 2005), which hinders translating *Drosophila* findings to mammals. In addition, some genes known to regulate vertebrate sleep lack clear invertebrate orthologs (Ferreira et al., 2017; de Lecea, 2021), while some genes that affect invertebrate sleep lack clear vertebrate orthologs (Koh et al., 2008; Toda et al., 2019). Finally, the sleep-wake cycle of *Drosophila*, with wakefulness peaks in the morning and evening and a mid-day siesta (Hendricks et al., 2000; Shaw et al., 2000), differs from the diurnal sleep-wake cycle of humans and some vertebrates, including zebrafish.

## Zebrafish as A Tool to Validate Candidate Disease Risk Genes Identified Using GWAS

A key strength of zebrafish is that technologies such as CRISPR/Cas9 (Hwang et al., 2013) and high-throughput behavioral assays (Prober et al., 2006; Thyme et al., 2019) allow large numbers of mutant lines to be generated and behaviorally tested efficiently and cost-effectively. Using zebrafish, one can systematically knock out and behaviorally phenotype each candidate gene within a genomic locus identified by GWAS in order to identify the causative gene. Indeed, a recent zebrafish screen used CRISPR/Cas9-induced targeted mutations to validate 132 candidate schizophrenia risk genes (Thyme et al., 2019) at 108 genomic loci that were identified by human GWAS (Schizophrenia Working Group of the Psychiatric Genomics, 2014). This targeted loss-of-function approach has also been used to characterize the swimming behavior of zebrafish containing mutations in the orthologs of 90 genes that have been associated with psychiatric disorders (Tang et al., 2020). Most recently, zebrafish were used to validate a candidate insomnia risk gene identified from a targeted *Drosophila* screen (Palermo et al., 2021) based on a human insomnia GWAS (Jansen et al., 2019). Automated annotation of the zebrafish genome found that over 70% of human genes have at least one zebrafish ortholog (Howe et al., 2013), and manual annotation suggests that this number is closer to 90% (Tran and Prober, unpublished observation). Thus, most human candidate genes will have at least one zebrafish ortholog.

## Behavioral Assays to Study Zebrafish Sleep

**Figure 2:**
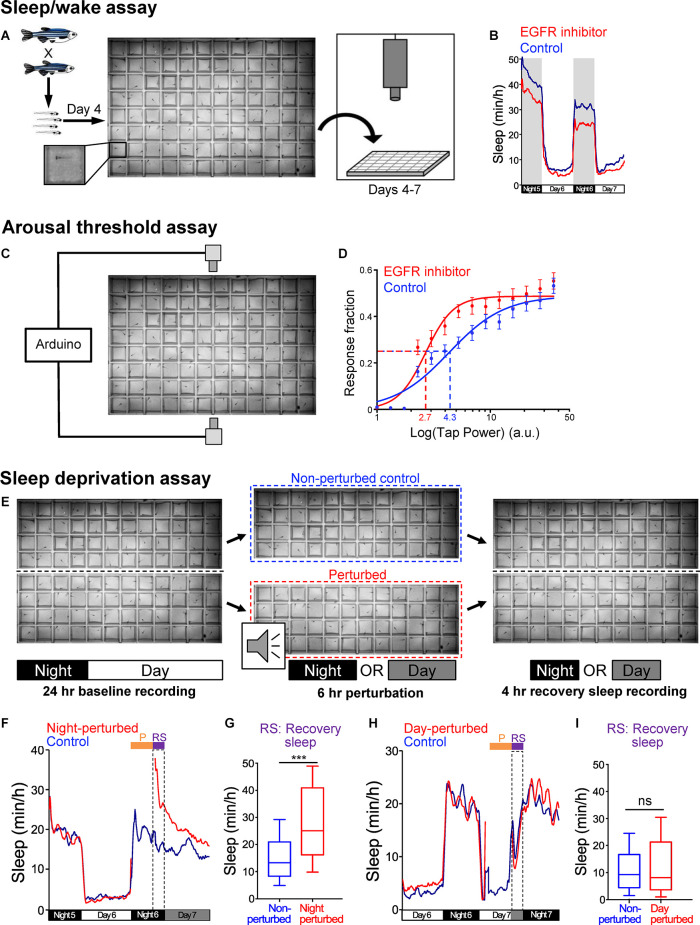
Zebrafish sleep assays. **(A)** Schematic of sleep assay. Larval zebrafish at 4-dpf obtained from an in-cross of heterozygous mutant fish are individually placed in each well of a 96-well plate that is filled with E3 medium and is maintained at 28.5°C by recirculating heated water. The plate is placed in a videotracking system, and is illuminated from below with white light from 9 a.m. to 11 p.m. and continuously with infrared light. The behavior of each fish is monitored by an infrared camera for up to 72 h. Animals are genotyped by PCR after the behavioral experiment is complete. **(B)** Example of data generated using the sleep assay. Zebrafish are diurnal, and thus sleep more at night compared to the day. Treatment of WT larval zebrafish with an EGFR small molecule inhibitor results in reduced sleep during both the night and day compared to siblings treated with DMSO vehicle control. **(C)** Schematic of arousal threshold assay. The videotracking system shown in **(A)** is modified by adding an Arduino board to control two solenoids that deliver a mechano-acoustic tapping stimulus to the 96-well plate during the night. The stimuli are applied over a range of intensities in random order at 1-min intervals while behavior is monitored. **(D)** Example of data generated using the arousal threshold assay. The fraction of animals that exhibit a startle response to each stimulus is plotted against the stimulus intensity (arbitrary units, a.u.) to generate a stimulus-response curve. The effective tap power 50 (ETP_50_) is the stimulus intensity that elicits 50% of the maximal response. Treatment of WT zebrafish with an EGFR small molecule inhibitor results in a decreased ETP_50_ (red dashed line) compared to siblings treated with DMSO vehicle control (blue dashed line), indicating that inhibition of EGFR results in a decreased arousal threshold. **(E)** Schematic of sleep deprivation assay. Larval zebrafish are placed in each well of a 96-well plate that is cut in half, and both halves of the plate are placed in the videotracking system shown in **(A)**. Behavior is first monitored for 24 h to quantify baseline sleep levels. On the second night, half of the plate is removed from the videotracker and is subjected to a complex mechano-acoustic perturbation in the dark for the first 6 h of the night, while the other half plate is left in the videotracker to serve as a non-perturbed control. The half-plate that is subjected to the perturbation is then returned to the videotracker, and the amount of recovery sleep is quantified by comparing the amount of sleep during the remaining 4 h of the night for the perturbed and non-perturbed animals. The amount of recovery sleep can also be normalized by comparison to the amount of sleep during the baseline night (not shown). **(F–I)** A sleep deprivation assay should result in increased sleep following perturbation during the normal sleep phase, but not following perturbation during the normal wake phase. Using this assay, there is an increase in sleep after the perturbation when it is applied at night **(F,G)** but not when it is applied during the day **(H,I)**. Fish are maintained in darkness (gray shading) after the perturbation in order to avoid the arousing effect of light on behavior. Dashed boxes indicate the 4 h period during which the amount of recovery sleep is quantified. ***P < 0.005, n.s. = not significant, by Mann-Whitney test. Figures are reproduced and modified from Prober et al. (2006) and Lee et al. (2019).

Sleep can be defined on the basis of three behavioral criteria (Joiner, 2016). First, sleep is associated with behavioral quiescence that is rapidly reversible, which distinguishes it from paralysis or coma. Second, sleep is associated with an increased arousal threshold, which distinguishes sleep from quiet wakefulness. Third, sleep is controlled by a homeostatic process, such that sleep-deprived animals exhibit increased sleep need. Using these criteria, sleep-like states have been described in several animal models, including fruit flies, nematodes, jellyfish, and zebrafish (Hendricks et al., 2000; Shaw et al., 2000; Zhdanova et al., 2001; Prober et al., 2006; Van Buskirk and Sternberg, 2007; Yokogawa et al., 2007; Raizen et al., 2008; Nath et al., 2017). Since the first description of zebrafish sleep (Zhdanova et al., 2001), *Danio rerio* has emerged as a useful animal model for vertebrate sleep, with a large body of evidence demonstrating conserved genetic, pharmacological, neuronal, and anatomical aspects of mammalian sleep (Barlow and Rihel, 2017; Levitas-Djerbi and Appelbaum, 2017; Oikonomou and Prober, 2017). Larval zebrafish behavior can be studied using a high-throughput automated videotracking system (Figure 2A). Individual larval zebrafish are placed in each well of a 96-well plate, and the behavior of each animal is recorded using an infrared camera (Prober et al., 2006). Starting at 5-days post-fertilization (dpf), zebrafish exhibit robust diurnal rest/activity cycles.

In order to measure arousal threshold, several assays that use visual or mechano-acoustic stimuli have been developed (Zhdanova et al., 2001; Prober et al., 2006; Elbaz et al., 2012; Singh et al., 2015). For example, one of these assays uses the same 96-well plate and automated videotracking system that is used to monitor locomotor activity, but subjects the animals to a tapping stimulus that is applied over a wide range of intensities, with multiple trials at each intensity, for several hours at night (Figure 2C; Singh et al., 2015). The stimuli are applied at 1-minute intervals, which is long enough to prevent habituation (Burgess and Granato, 2007; Woods et al., 2014). By quantifying the fraction of animals that show a behavioral response at each stimulus intensity, the stimulus intensity that elicits 50% of the maximal response (effective tap power 50, ETP_50_) can be calculated and serves as a measure of arousal threshold. Behavioral responses to these stimuli can also be used to identify differences in sleep depth by comparing the fraction of sleeping animals of different genotypes that respond to the stimulus (Oikonomou et al., 2019). Assays that use different stimuli have all found that larval zebrafish show a large increase in arousal threshold following 1 minute of inactivity, indicating that 1 or more minutes of rest corresponds to a sleep-like state in larval zebrafish (Prober et al., 2006; Elbaz et al., 2012; Singh et al., 2015).

Several assays have been developed to measure sleep homeostasis in both larval (Zhdanova et al., 2001; Lee et al., 2019; Leung et al., 2019; Oikonomou et al., 2019; Reichert et al., 2019) and adult (Yokogawa et al., 2007) zebrafish. For example, one assay uses a 96-well plate that is cut in half (Figure 2E; Lee et al., 2019). After a night of baseline sleep is recorded using the videotracking system, the animals in one half-plate are subjected to a complex mechano-acoustic stimulus during the first 6 hours of the night, while animals in the other half-plate are not perturbed and serve as controls. Sleep rebound is then quantified by comparing the amount of sleep during the remaining 4 hours of the night between perturbed and non-perturbed animals. Importantly, sleep rebound is only observed when animals are perturbed at night and not when they are perturbed during the day (Figures 2F–I), indicating that rebound sleep is due to loss of sleep rather than other factors such as stress or fatigue.

## Advantages of Zebrafish as An Animal Model for Sleep Research

The zebrafish has several features that make it a useful sleep model. Due to their small size and robust sleep/wake cycles that appear as early as 5-dpf, larval zebrafish are amenable to high-throughput screens that have identified genes (Chiu et al., 2016) and drugs (Rihel et al., 2010) that affect sleep. Indeed, screens have shown that most drugs that affect mammalian sleep have similar effects on larval zebrafish (Renier et al., 2007; Rihel et al., 2010), suggesting that similar mechanisms underlie mammalian and zebrafish sleep. Due to their small size, many larval zebrafish can be tested in parallel in 96-well plates, which provides sufficient statistical power to detect even subtle sleep phenotypes. The larval zebrafish brain shares many anatomical similarities with that of mammals (Tropepe and Sive, 2003), including structures thought to be most important for mammalian sleep, such as the hypothalamus and hindbrain, as well as several neuropeptidergic and neuromodulatory populations (Barlow and Rihel, 2017; Levitas-Djerbi and Appelbaum, 2017; Oikonomou and Prober, 2017). Indeed, neuropeptides and neuromodulators that affect mammalian sleep have similar functions in zebrafish (Prober et al., 2006; Singh et al., 2015; Chen A. et al., 2016, Chen S. et al., 2016, Chen S. et al., 2017; Chiu et al., 2016; Leung et al., 2019; Oikonomou et al., 2019; Reichert et al., 2019). There is also evidence that zebrafish sleep may be associated with brain oscillations that are similar to those observed during mammalian sleep (Leung et al., 2019). However, the larval zebrafish brain has >100 fold fewer neurons than the rodent brain, thus providing a more tractable system to study basic neuronal mechanisms that underlie vertebrate sleep. Zebrafish are also diurnal (Hurd et al., 1998), and thus may be a better model than commonly used nocturnal rodents for the circadian regulation of human sleep. Indeed, genetic studies in zebrafish provided long-sought proof that melatonin is required for circadian regulation of sleep in a diurnal vertebrate animal (Gandhi et al., 2015). Due to its transparency at embryonic and larval stages, zebrafish are amenable to non-invasive optogenetic stimulation of genetically specified neurons (Singh et al., 2015) and whole-brain neuronal activity monitoring (Ahrens et al., 2013) that can be used to identify and manipulate specific neuronal populations in behaving animals (Del Bene and Wyart, 2012; Singh et al., 2015; Orger and de Polavieja, 2017; Lee et al., 2020). Genetic techniques such as CRISPR/Cas9 mutagenesis (Hwang et al., 2013) and *Tol2* transgenesis (Kawakami et al., 2000) allow rapid and cost-effective generation of mutant and transgenic lines.

Finally, there are several examples where mutation of a gene in rodents results in embryonic or perinatal lethality, but knocking out the zebrafish ortholog results in a sleep phenotype with no apparent developmental defects. As a result, it has been possible to use genetics to study the roles of some genes in sleep using zebrafish that has not been possible using rodents. For instance, mutation of *dopamine beta hydroxylase* (*dbh*), which is required for the synthesis of noradrenaline, is embryonic lethal in mice, while zebrafish *dbh* mutants show a large increase in sleep but no developmental defects (Singh et al., 2015). It is unclear why some genes are required for rodent but not zebrafish development, but this may be due in part to challenges of development *in utero* for rodents compared to *ex utero* for zebrafish, as well as the dependence of newborn rodents, but not zebrafish, on their mother for survival. Another example is melatonin, whose role in mammalian sleep has not been evaluated using genetics because most laboratory mouse strains produce little or no melatonin. This lack of melatonin is due to the mutation of genes that are required for melatonin synthesis (Goto et al., 1989), likely due to selection pressures in the laboratory setting. Thus, the use of zebrafish genetics has facilitated the discovery of important mechanisms that regulate sleep that has not been possible using rodents.

## Limitations of Zebrafish as An Animal Model for Sleep Research

Despite numerous advantages of zebrafish for studying sleep, it has several limitations as a sleep model. Mammalian sleep is often defined based on recordings of electrical activity in the brain (electroencephalogram, EEG) and muscle (electromyogram, EMG) that can precisely define sleep and wake states. Although methods for non-invasive EEG recordings in larval (Hong et al., 2016) and adult (Cho et al., 2017) zebrafish have been developed, these methods have only been used to monitor large changes in brain activity that occur during seizures. However, several studies have shown that mammalian sleep can be studied using behavioral measures alone, similar to zebrafish. For example, behavioral assays that measure sleep duration in mice have been developed that have a high correlation with EEG/EMG measures of sleep (Pack et al., 2007; Fisher et al., 2012). Similarly, sleep in humans has been measured using actigraphy, with results similar to those obtained using EEG/EMG (Ancoli-Israel et al., 2003; Van de Water et al., 2011; Yetish et al., 2015). A limitation of this approach is that when sleep is defined using behavioral measures, it can be difficult to distinguish sleep from direct effects on the motor system. However, sleep is rapidly reversible and is associated with an increased arousal threshold, thus distinguishing it from paralysis. Another limitation of zebrafish is that it is more distantly related to humans than rodents, and thus rodents may provide a better model for genetic regulation of human sleep. However, as noted above, most human genes have at least one zebrafish ortholog, and most genes that affect mammalian sleep have similar effects on zebrafish behavior (Barlow and Rihel, 2017; Levitas-Djerbi and Appelbaum, 2017; Oikonomou and Prober, 2017). A final concern is that zebrafish lack some mammalian brain structures, such as a large cerebral cortex, that may play roles in sleep (Krone et al., 2021). However, the zebrafish dorsal telencephalon has similarities to mammalian cortical regions based on molecular markers (Wullimann and Mueller, 2004; Mueller et al., 2011) and functional assays (Aoki et al., 2013; Lal et al., 2018; Torigoe et al., 2021).

## Generating Zebrafish Mutants Based on Candidate Human Sleep Disorder Risk Genes

**Figure 3:**
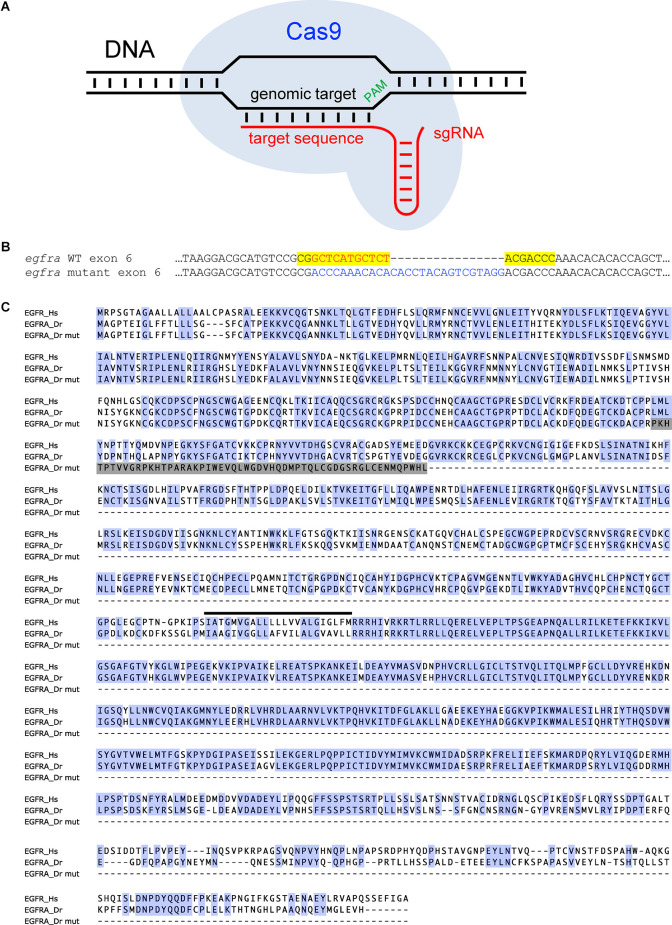
Using CRISPR/Cas9 to generate a predicted null mutation in the zebrafish *egfra* gene. **(A)** A schematic diagram of the CRISPR/Cas9 system. The Cas9/sgRNA complex targets a specific sequence in the genome based on a 20 base-pair target sequence in the sgRNA that is complementary to the genomic target next to a protospacer adjacent motif (PAM) and produces a double-stranded DNA break. **(B)** The coding sequence of a region of exon 6 from WT and mutant zebrafish *egfra* are shown. The mutant contains an 11 bp deletion (red font) and a 27 bp insertion (blue font) in the *egfra* gene. Yellow shading indicates the sgRNA target sequence. **(C)** An alignment of human EGFR (Hs), zebrafish WT EGFRA (Dr), and zebrafish mutant EGFRA (Dr mut) proteins is shown. Blue shading indicates amino acids that are identical in the human and zebrafish orthologs. Gray shading indicates frame shifted sequence in the zebrafish EGFRA mutant. The black line indicates the location of the transmembrane domain in the WT proteins. The mutant protein lacks the transmembrane domain and intracellular domains that are required to interact with downstream effector proteins, and thus should be non-functional. Reproduced and modified from Lee et al. (2019).

To perform a targeted screen for genes whose loss affects sleep, genome-editing techniques such as CRISPR/Cas9 (Hwang et al., 2013; Figure 3A) can be used to generate a large collection of zebrafish lines, each containing a predicted null mutation in the zebrafish ortholog of a candidate human sleep disorder risk gene. Tools such as CHOPCHOP (Labun et al., 2016, 2019) and CRISPRscan (Moreno-Mateos et al., 2015) can be used to identify single guide RNAs (sgRNAs) with high predicted activity and no predicted off-target sites in the genome. Ideally, one should select sgRNAs that target exons between or within domains that are essential for protein function. To generate mutants, an sgRNA and Cas9 mRNA (Hwang et al., 2013) or Cas9 protein (Jao et al., 2013) are microinjected into embryos at the 1-cell stage, and sgRNAs with high cleavage efficiency can be identified using a variety of methods, including T7 endonuclease assay (Hua et al., 2017), high-resolution melting curve analysis (Thomas et al., 2014), or high-throughput sequencing (Gagnon et al., 2014). Once an effective sgRNA is identified, embryos are injected with that sgRNA and Cas9 mRNA or protein and are raised to adulthood. These F_0_ fish are then outcrossed to wild-type (WT) fish, and their F_1_ progeny are screened for mutations at the embryonic or larval stage of development using PCR fragment analysis (Varshney et al., 2016) or other methods (Sentmanat et al., 2018). Once F_0_ fish carrying germline mutations are identified, they are again outcrossed to WT fish to generate F_1_ animals, which are raised to adulthood and genotyped by PCR to identify heterozygous mutant carriers. The specific nature of each mutation can then be determined by Sanger sequencing. Usually, mutants are selected that contain an insertion/deletion (indel) mutation that is predicted to cause a shift in the translational reading frame, since this changes the protein sequence and often introduces a premature stop codon, thus generating a truncated protein that lacks essential functional domains. Alternatively, the first exon or an entire gene can be deleted by co-injecting two sgRNAs that flank the region to be deleted (Chen et al., 2014), or sequences containing a stop codon can be inserted into the coding sequence of a gene (Gagnon et al., 2014), but these methods are less efficient than generating indel mutations. Ideally, two independent mutant lines should be established for each candidate gene in order to increase confidence that any phenotype is due to mutation of the gene of interest. Mutants should be outcrossed to the parental WT strain for at least two generations before behavioral assays are performed to minimize the presence of any off-target mutations that might affect behavior.

In order to reduce the amount of time and effort needed to generate and characterize mutant animals, several studies have proposed performing screens of somatic mutations in F_0_ injected embryos and larvae (Wu et al., 2018; Hoshijima et al., 2019; Kroll et al., 2021; Quick et al., 2021). This strategy involves injecting Cas9 protein along with multiple sgRNAs that target different regions of the same gene, with scrambled sgRNAs used as negative controls. This approach has been shown to work well when testing for developmental phenotypes such as loss of pigmentation (Kroll et al., 2021), loss of specific neuronal populations (Liu et al., 2015), and eye defects (Hoshijima et al., 2019). In proof-of-concept experiments, the potential utility of this approach to generate behavioral phenotypes was demonstrated by assaying behavioral responses to a chemical irritant and changes in locomotor activity in larval zebrafish (Kroll et al., 2021). Despite the efficiency of this strategy, it is unclear whether it will be useful for identifying sleep mutants for several reasons. First, in F_0_ injected embryos and larvae, the targeted gene is only knocked out in a subset of cells in an animal, and the number of such cells will vary among injected animals. Since sleep experiments require that behavioral data from many animals be analyzed as a group, and it is difficult to determine whether the targeted gene is knocked out in the relevant cells in each animal, phenotypes in F_0_ injected animals are likely to be smaller and more variable compared to those in germline mutants, where the targeted gene is mutated in every cell of an animal. This caveat is less of a problem for developmental assays, where mutant phenotypes can be detected in individual animals, including animals that are mosaic for mutant cells, and one can often directly observe mutant and WT cells in an animal (Liu et al., 2015; Kroll et al., 2021). Second, while computational tools are used to select sgRNAs that are predicted to lack off-target sites in the genome (Moreno-Mateos et al., 2015; Labun et al., 2016, 2019), co-injecting multiple sgRNAs nevertheless increases the likelihood of generating off-target mutations (Chen et al., 2014; McCarty et al., 2020) which can affect behavior. Third, the microinjection procedure can cause non-specific developmental defects that affect behavior but are too subtle to detect by visual inspection. This can result in fish that have abnormal behavior, which in some cases can be difficult to distinguish from sleep phenotypes. Indeed, the behavior of microinjected fish can be more variable than that of uninjected controls (Chiu et al., 2016), and robust sleep phenotypes observed in germline mutants may not be apparent in F_0_ microinjected fish.

## Concerns with Generating and Screening Zebrafish Mutants

A potential problem with using genetic approaches to study sleep is that mutants may have developmental defects that preclude or confound behavioral testing. Indeed, this has been a significant challenge for attempts to use genetics to study mammalian sleep (Thomas et al., 1995; Alenina et al., 2009). It is estimated that 1,400–2,500 zebrafish genes are essential for embryonic development (Driever et al., 1996; Haffter et al., 1996; Amsterdam et al., 2004), and there are likely many additional genes whose loss results in animals that are viable but have developmental defects. Thus, mutants must be carefully screened for developmental defects prior to testing them for sleep phenotypes. Even if a mutant does not show obvious developmental phenotypes, it may have subtle developmental defects or be developmentally delayed, which can result in phenotypes that may be difficult to distinguish from effects on sleep. This risk can be mitigated by testing mutants for muscle and motor defects by light touch with a blunt needle and by tapping the dish containing the animals, both of which should elicit a startle response. Ideally, mutant phenotypes should be confirmed using acute perturbations where effects on development are less of a concern, such as acute treatment with a drug that inhibits the targeted protein or its signaling pathway, or by targeted ablation of cells that express the protein. In addition, showing that an acute gain-of-function perturbation results in a phenotype opposite to that of the mutant increases confidence that a mutant phenotype is due to effects on behavior and not on development. This can be achieved using several approaches, including heat shock-induced overexpression of the gene of interest (Prober et al., 2006), and optogenetic (Singh et al., 2015) or chemogenetic (Chen S. et al., 2016) stimulation of neurons that express the gene of interest.

An important consideration when using loss-of-function mutations in zebrafish is that the teleost lineage underwent genome duplication events after its divergence from the mammalian lineage, and at least 20% of duplicated genes have been retained (Postlethwait et al., 2000). As a result, the zebrafish genome contains two or more paralogs of some human genes, which can have both advantages and disadvantages for genetic studies. One advantage is that some genes that are required for mammalian development or whose loss results in phenotypes that confound sleep analyses can be studied in zebrafish if the two zebrafish paralogs are at least partially redundant. For example, mutation of *ATPase Na+/K+ transporting subunit alpha 3 (ATP1A3)* in humans is associated with rapid-onset dystonia-Parkinsonism (de Carvalho Aguiar et al., 2004) and alternating hemiplegia of childhood (Heinzen et al., 2014). *atp1a3* mutant mice exhibit seizures (Clapcote et al., 2009) and motor deficits (Kirshenbaum et al., 2011) which complicate the study of sleep. Zebrafish have two *atp1a3* paralogs (*atp1a3a* and *atp1a3b*), and while fish that lack both paralogs apparently die during embryogenesis, loss of either gene alone has no apparent effect on development or gross motor functions. However, *atp1a3a*, but not *atp1a3b*, mutant zebrafish show a large reduction in sleep at night (Barlow et al., 2020). Thus, the study of *atp1a3* in zebrafish revealed a function in sleep that would be difficult to detect in mammals. Another advantage arises when multiple zebrafish paralogs of a single mammalian gene are expressed in different cell populations, thus allowing the function of each population to be studied separately. For example, zebrafish have three paralogs of mammalian *tryptophan hydroxylase (tph)*, the rate-limiting enzyme in the biosynthesis of serotonin. In zebrafish, *tph1a* is expressed in the retina, pineal gland, preoptic area, posterior tuberculum, and caudal hypothalamus, whereas *tph1b* is only transiently expressed in preoptic neurons of the hypothalamus during development (Bellipanni et al., 2002). In contrast, although *tph2* is expressed in the pineal gland, similar to *tph1a*, it is also expressed in serotonergic raphe neurons, which do not express *tph1a* or *tph1b* (Teraoka et al., 2004). Mutation of *tph2* and manipulation of *tph2*-expressing neurons revealed that serotonergic raphe neurons promote sleep, thus resolving a longstanding controversy in the field (Oikonomou et al., 2019).

A disadvantage of having multiple zebrafish paralogs of single mammalian genes is that if the paralogs are redundant, it is necessary to knock out both genes in order to observe a phenotype. For example, human *gpr103*, which acts as a receptor for the neuropeptide QRFP (Takayasu et al., 2006), has two paralogs in zebrafish (*gpr103a* and *gpr103b*; Chen A. et al., 2016). While mutation of either paralog has no effect on sleep in zebrafish, double mutants sleep less during the day compared to their WT siblings (Chen A. et al., 2016). Another disadvantage arises when a mutation causes nonsense-mediated decay (NMD) of the mutated gene’s mRNA, which often occurs for indel mutations that are generated using CRISPR/Cas9. NMD can trigger a compensatory response in which the expression of paralogs and other related genes are upregulated, thus potentially masking a mutant phenotype (Rossi et al., 2015; Anderson et al., 2017; El-Brolosy et al., 2019). The presence of NMD can be determined by performing RT-qPCR or *in situ* hybridization using a probe specific for the targeted gene and comparing its expression level in homozygous mutant animals to their WT siblings. If NMD is observed, and particularly if there is no mutant phenotype, other mutant alleles can be generated by using sgRNAs that target other regions of the gene, which may create mutations that do not result in the induction of NMD. Alternatively, NMD can be avoided by deleting the gene of interest from the genome by using a pair of sgRNAs that flank the coding sequence (El-Brolosy et al., 2019). However, this strategy will remove introns that might contain sequences that affect the expression of other genes, and thus deletion of only the first exon may be preferable. Ideally, loss of the targeted protein should be evaluated using an antibody that is specific for the mutated gene, although antibodies are not available for most zebrafish proteins.

## Screening Mutants for Sleep Phenotypes and Integrating Validated Sleep Disorder Risk Genes into Known Pathways

To test zebrafish mutants for sleep defects, adult heterozygous mutant animals are in-crossed to generate homozygous mutant, heterozygous mutant, and WT sibling larvae. Individual 4-dpf fish are placed in each well of a 96-well plate filled with E3 medium and maintained at 28.5°C. A videotracking system (Figure 2A) is used to monitor the behavior of each animal for up to 72 hours, during which animals are exposed to white light from 9 a.m. to 11 p.m., while infrared light and an infrared camera are used to record behavior. Each animal is then genotyped by PCR to identify homozygous mutant, heterozygous mutant, and homozygous WT siblings. MATLAB scripts are used to quantify the duration of locomotor activity and sleep, the length and number of wake and sleep bouts, the latency to first sleep bout at night, and the amount of locomotor activity while awake. This method has been used for zebrafish behavioral screens (Rihel et al., 2010; Chiu et al., 2016; Thyme et al., 2019) and to characterize sleep phenotypes in mutant zebrafish (Gandhi et al., 2015; Singh et al., 2015, 2017; Yelin-Bekerman et al., 2015; Chen A. et al., 2016, Chen A. et al., 2017, Chen S. et al., 2017; Chiu et al., 2016; Hoffman et al., 2016; Lee et al., 2017, 2019; Ashlin et al., 2018; Leung et al., 2019; Oikonomou et al., 2019; Reichert et al., 2019; Levitas-Djerbi et al., 2020; Özcan et al., 2020; Kroll et al., 2021). This behavioral assay has also been modified to measure habituation to acoustic stimuli, prepulse inhibition, and behavioral responses to light, darkness, heat, and noxious chemicals (Prober et al., 2008; Chen S. et al., 2017; Thyme et al., 2019).

In a recent proof-of-concept study, GWAS analyses implicated the epidermal growth factor receptor (EGFR) signaling pathway in regulating sleep, a finding that was then validated using zebrafish (Lee et al., 2019). A human GWAS sleep trait analysis identified genome-wide significant signals near *ERBB4* (Lee et al., 2019) and *KSR2* (Figure 1B), which are known to participate in the EGFR signaling pathway (Cohen et al., 1996; Dhawan et al., 2016). Consistent with this observation, a separate GWAS analysis found that associations at the EGFR signaling pathway are enriched for daytime sleepiness in humans (Wang et al., 2019). To validate a role for EGFR signaling in sleep, zebrafish containing loss-of-function mutations in the zebrafish orthologs of *epidermal growth factor receptor a* (*egfra*; Figures 3B,C), or the EGFR ligands *transforming growth factor alpha* (*tgfa*) and *epidermal growth factor* (*egf*), resulted in decreased sleep, as did acute pharmacological inhibition of EGFR (Figure 2B), ERBB4, or KSR2 (Lee et al., 2019). Conversely, overexpression of *tgfa* resulted in increased sleep in an *egfra*-dependent manner. Arousal threshold and sleep deprivation assays revealed that EGFR signaling regulates both arousal threshold (Figure 2D) and sleep homeostasis (Lee et al., 2019). These results indicate that EGFR signaling functions to promote sleep in zebrafish, consistent with previous studies showing that EGFR signaling promotes sleep in *Drosophila* (Foltenyi et al., 2007; Donlea et al., 2009; Harbison et al., 2013, 2017) and *C. elegans* (Van Buskirk and Sternberg, 2007; Hill et al., 2014; Nelson et al., 2014; Nath et al., 2016). Thus, EGFR signaling has an evolutionarily conserved function to promote sleep.

Once a role for EGFR signaling in zebrafish sleep was established, further work integrated this finding into a sleep-regulating mechanism that had been discovered by animal model studies. A previous study found that the hypothalamic neuropeptide VF (NPVF) promotes sleep in zebrafish (Lee et al., 2017), similar to EGFR signaling. When a potential link between these pathways was explored, it was found that EGFR signaling promotes both the expression of *npvf* and the activity of *npvf*-expressing neurons, and genetic epistasis experiments indicated that NPVF acts downstream of EGFR to promote sleep (Lee et al., 2019). Another study showed that NPVF signaling promotes sleep *via* the serotonergic raphe nuclei in zebrafish (Lee et al., 2020), which have a sleep-promoting function in both zebrafish and mice (Oikonomou et al., 2019). A sleep-promoting function for the serotonergic raphe in zebrafish and mice is consistent with human GWAS, which found that variants in genes participating in serotonergic signaling are enriched for association with human sleep traits (Lane et al., 2017, 2019; Dashti et al., 2019; Jones et al., 2019). These studies illustrate how human GWAS and animal model studies can be used to identify genes that underlie human sleep disorders and to determine the genetic and neuronal mechanisms through which these genes affect sleep.

## Conclusion

Since the first GWAS was published 20 years ago (Ozaki et al., 2002), hundreds of thousands of genomic risk loci associated with thousands of human traits and diseases have been identified (MacArthur et al., 2017; Loos, 2020). This work has led to the development of comprehensive GWAS databases containing information on identified disease/trait-associated variants and nearby genes (Buniello et al., 2019; Wang et al., 2020). However, a bottleneck currently exists in the development of tools and animal models to identify the causative gene that is associated with each genomic risk locus. The use of rodent models is limited by the challenges of generating, maintaining, and testing large numbers of mutant lines, while zebrafish provide a useful alternative vertebrate model to validate human candidate disease risk genes efficiently and cost-effectively, and to then perform mechanistic studies to understand the involvement of each gene in disease. Indeed, zebrafish have been used to validate human candidate genes associated with insomnia (Palermo et al., 2021), schizophrenia (Thyme et al., 2019), diabetes (Adeyemo et al., 2019), abnormal liver function (Liu et al., 2013), idiopathic scoliosis (Mathieu et al., 2021), attention deficit hyperactivity disorder (ADHD; Dark et al., 2020), and other psychiatric disorders (Tang et al., 2020). The strategies described in this review are not limited to GWAS, but can also be applied to other disease gene discovery strategies. For example, whole-exome and whole-genome sequencing of individuals with neuropsychiatric disorders are increasingly being used to identify candidate disease risk genes (Sanders et al., 2017), but similar to GWAS it is often unclear whether any specific candidate gene is causative for disease. This is particularly the case for non-coding variants, which represent over 90% of disease-associated genomic risk loci identified through GWAS (Sanders et al., 2017). Thus, similar to GWAS, candidate disease risk genes identified using sequencing often require validation studies in an animal model. Zebrafish models have already been useful for this purpose, for example validating *nr3c2* as an autism spectrum disorder risk gene based on human whole-genome sequencing and zebrafish functional assays (Ruzzo et al., 2019). Thus, the use of zebrafish models has the potential to accelerate the identification of disease risk genes, aid in the acquisition of mechanistic insights, and inform the development of new therapies.

## Author Contributions

ST and DAP both contributed to the writing of this manuscript. All authors contributed to the article and approved the submitted version.

## Conflict of Interest

The authors declare that the research was conducted in the absence of any commercial or financial relationships that could be construed as a potential conflict of interest.

## Publisher’s Note

All claims expressed in this article are solely those of the authors and do not necessarily represent those of their affiliated organizations, or those of the publisher, the editors and the reviewers. Any product that may be evaluated in this article, or claim that may be made by its manufacturer, is not guaranteed or endorsed by the publisher.
